# Smac mimetic LCL161 supports neuroblastoma chemotherapy in a drug class-dependent manner and synergistically interacts with ALK inhibitor TAE684 in cells with ALK mutation F1174L

**DOI:** 10.18632/oncotarget.12055

**Published:** 2016-09-15

**Authors:** Safiullah Najem, Doerte Langemann, Birgit Appl, Magdalena Trochimiuk, Patrick Hundsdoerfer, Konrad Reinshagen, Georg Eschenburg

**Affiliations:** ^1^ Department of Pediatric Surgery, University Medical Center Hamburg-Eppendorf, Hamburg, Germany; ^2^ Department of Pediatric Oncology/Hematology, Charité - Universitätsmedizin Berlin, Berlin, Germany

**Keywords:** neuroblastoma, LCL161, Smac mimetics, chemotherapy, anaplastic lymphoma kinase

## Abstract

Neuroblastoma is the most common extracranial solid tumor during infancy and childhood.

Outcome of high-risk and late-stage disease remains poor despite intensive treatment regimens.

Suppressing inhibitor of apoptosis proteins (IAPs) using Smac mimetics (SM) significantly sensitizes neuroblastoma (NB) cells for chemotherapy, however strongly dependent on the cytotoxic drug combined with SM.

Therefore, a systematic analysis of the impact of SM in combination with different classes of chemotherapeutics was of crucial importance. Treatment of NB cell lines with SM LCL161 and vinca alkaloids revealed a strong synergistic inhibition of proliferation and significant induction of apoptosis in virtually all established and *de novo* NB cell lines (*n*=8).

In contrast, combination of anthracyclines or topoisomerase inhibitors with LCL161 showed a synergism for single drugs and/or cell lines only.

Furthermore, we could show that insensibility to LCL161-mediated sensitization for chemotherapeutics is associated with aberrant activation of anaplastic lymphoma kinase (ALK) by common mutation F1174L. Inhibition of ALK using TAE684 is able to overcome this resistance in a synergistic fashion, a finding that could be highly relevant for improvement of neuroblastoma therapy.

## INTRODUCTION

Neuroblastoma is a malignant disease of the sympathetic nervous system and the most prevalent solid extracranial tumor during infancy and childhood. Prognosis for non-high risk disease is good with 5-year survival > 90%, at the same time more than 50% of patients suffer from metastatic disease at time of diagnosis [1, 2]. Outcome for these patients is still poor in spite of significant therapy improvements in the last years including differentiation therapy or immunotherapy [3]. Development of novel treatment options is therefore absolutely essential and a key objective in pediatric oncology [4, 5].

Apoptosis is a form of programmed cell death that is necessary to eradicate impaired cells and to enable realization of developmental programs. Deregulated apoptotic machinery is one of the hallmarks of cancer contributing to development and expansion of diverse malignancies [6].

The family of inhibitor of apoptosis proteins (IAP) are essential regulators of apoptosis. X-linked IAP (XIAP), the most prominent and best-analyzed IAP, is increased in different cancers including neuroblastoma contributing to chemotherapy resistance and unfavorable outcome [7-10]. Caspase activation essential for apoptosis induction is inhibited by direct binding of XIAP to caspases-3/−7/−9 [11]. Second mitochondria-derived activator of caspase (Smac) is released from the mitochondria in response to different apoptotic stimuli. IAPs are antagonized by interaction of their BIR2 and BIR3 domains with the AVPI tetrapeptide motif of Smac [12]. Using this motif as a blue print for the development of small molecule IAP antagonists (Smac mimetics) has shown to be a promising strategy for treatment of malignancies associated with XIAP overexpression.

Smac mimetics (SM) were able to induce apoptosis and to significantly increase efficacy of classic treatment in several experimental cancer models [10, 13]. In neuroblastoma cell lines we could demonstrate a sensitization against cytotoxic drugs currently used in neuroblastoma therapy *in vitro* and in a syngeneic mouse model using SM LBW242 and LCL161 [7]. LBW242 and LCL161 are structural analogs with similar molecular weight; the latter was optimized for potency and pharmacokinetics [14]. Several clinical trials are currently conducted to evaluate the capacity of Smac mimetics for the treatment of resistant malignant diseases [15].

Sensitization of neuroblastoma cells for chemotherapy using SM was highly synergistic for vincristine, in part synergistic for doxorubicin and antagonistic for etoposide [7].

If the synergistic interaction of SM is drug-dependent or related to the molecular background of the neuroblastoma cells is still unanswered.

Therefore, a systematic effect analysis of different classes of antineoplastic drugs in combination with SM was the logical next step to shed light on this relevant issue. In the current study, we could show that in all except one of the tested neuroblastoma cell lines vinca alkaloids (vinblastine, vindesine and vincristine) with SM LCL161 displayed a strong synergistic effect on proliferation and a significant apoptosis induction in line with the results obtained before. Using anthracyclines (daunorubicin, doxorubicin and idarubicin) or topoisomerase inhibitors (etoposide, topotecan and SN-38) in contrast a synergism with LCL161 was detectable for single drugs and/or cell lines only.

## RESULTS

Smac mimetic LBW242 displayed a synergistic gain of chemotherapy on neuroblastoma cell lines in a drug-dependent manner [7]. Different classes of chemotherapeutics used in neuroblastoma therapy were thus selected for a systematic analysis of their combination effect with Smac mimetics (SM). LCL161, a structural analog of LBW242, was used for this survey because it is well tolerated in humans and mice [16, 17], and showed synergistic cooperation with vincristine in neuroblastoma *in vivo* as well [7]. Furthermore, LCL161 is currently evaluated in several clinical trials (www.clinicaltrials.gov).

### Protein expression of XIAP and cIAP-1 and influence on cellular proliferation and apoptosis following LCL161-treatment in neuroblastoma

**Figure 1:**
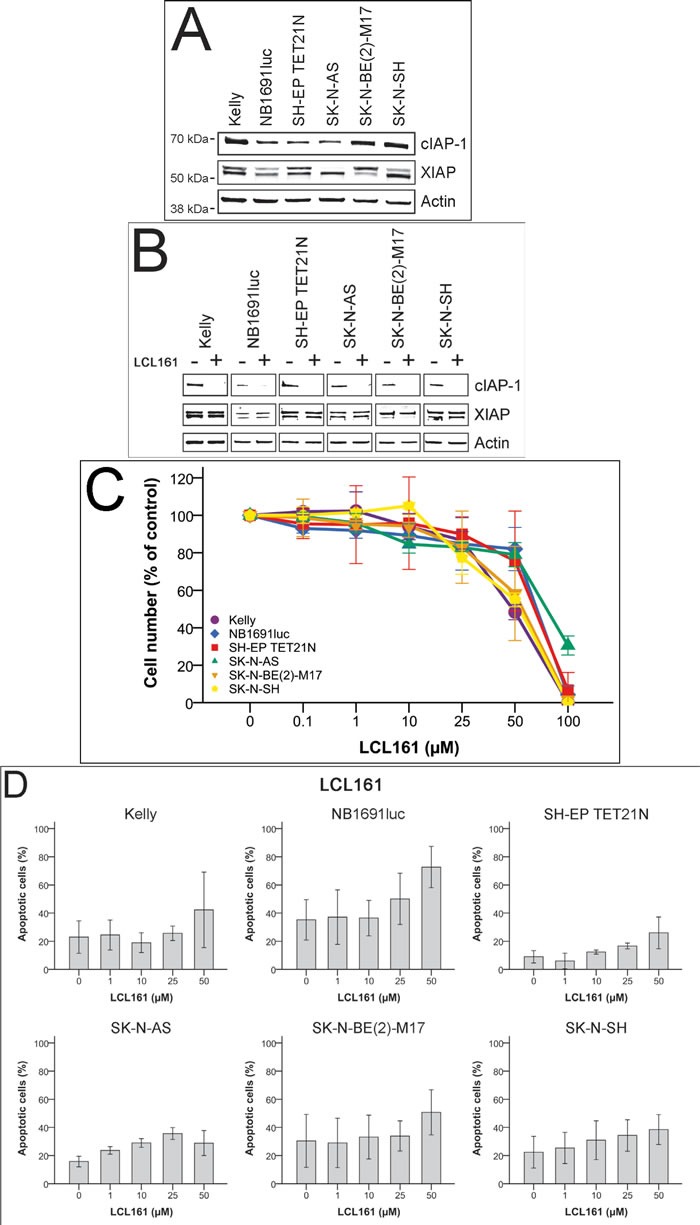
Effect of LCL161 on expression of cIAP-1 and XIAP, cell proliferation and induction of apoptosis in neuroblastoma cell lines Expression of cIAP-1, XIAP and actin was detected by Western Blot analysis in untreated neuroblastoma cell lines **A.** and cells treated 30 min with 10 μM LCL161 **B.**. Proliferation of cells treated with the indicated concentrations of LCL161 was determined after 48 h **C.**. Proliferation of untreated cells was defined as 100%. The proportion of apoptotic cells was determined by flow cytometry (Annexin V and PI staining) 48 h after LCL161 treatment **D.**. Values represent the mean ± SD of three independent experiments.

Apoptosis induction by SM is thought to be directly correlated to their binding and inactivation of XIAP and degradation of cIAP-1 respectively [10, 18-20]. Therefore we determined the abundance of cIAP-1 and XIAP protein in neuroblastoma cell lines (*n* = 6) using Western blot analysis (Figure 1A). Slight differences in expression levels for cIAP-1 and two specific XIAP protein bands were observed. We have demonstrated cIAP-1 degradation and constant XIAP expression in neuroblastoma cells following treatment with SM LBW242 [7]. Treatment of cells using LCL161 for 30 min showed a similar picture (Figure 1B).

Then we evaluated proliferation and apoptosis induction of human neuroblastoma cell lines (*n* = 6) in the presence of SM LCL161 monotherapy. Significant inhibition of proliferation was detected using high micromolar concentrations with IC_50_ of 49.4-77.9 μM (Figure 1C and Table 1A). For all following experiments concerning combinations of LCL161 with chemotherapeutic drugs a concentration of 10 μM LCL161 was used. With this concentration only marginal effects on proliferation and apoptosis induction of the tested neuroblastoma cell lines were observed (Figure 1D).

**Table 1:** Established and *de novo* neuroblastoma cell lines are sensitized by LCL161 for chemotherapy-induced inhibition of cell proliferation

Table 1A																			
	VBL IC_50_ (nM)		VCR IC_50_ (nM)		VDS IC_50_ (nM)		DAU IC_50_ (μM)		DOX IC_50_ (μM)		IDA IC_50_ (nM)		ETO IC_50_ (μM)		SN-38 IC_50_ (μM)		TOPO IC_50_ (μM)		LCL161 IC_50_ (μM)
LCL161 (10 μM)	-	+	-	+	-	+	-	+	-	+	-	+	-	+	-	+	-	+	
**Kelly**	16	5.4	79	20	48	20	.38	.29	.84	.65	74	72	4.6	3.1	.04	.03	.08	.08	49
**NB1691luc**	3.7	1.4	25	1.5	9.1	0.9	1.6	.80	2.6	.24	158	85	23	22	.35	.22	1.2	1.1	69
**SH-EP TET21N**	13	4.1	37	18	26	13	.61	.36	.6	.41	172	171	24	23	1.6	1.3	1	0.8	73
**SK-N-AS**	5.6	4.2	42	20	19	6.3	.37	.20	1.4	.35	223	110	27	20	.53	.38	.78	.22	78
**SK-N-BE(2)-M17**	7.9	6.2	117	110	121	34	.44	.27	.24	.11	56	45	29	24	.05	.03	.57	.48	59
**SK-N-SH**	5.6	3.9	79	7.1	58	12	.35	.23	.12	.05	42	34	.54	.44	.01	.01	.08	.07	56
**HGW-1**	6.4	2.2	22	4.7	16	4.2	.09	.07	.41	.15	48	47	4.9	1.8	.04	.03	.73	.27	34
**HGW-3**	12	2.1	33	4.7	39	4.5	.06	.04	.04	.02	29	26	7.5	1.8	.05	.04	.02	.02	71

Table 1B									
	VBL + LCL161 CI(IC_50_)	VCR + LCL161 CI(IC_50_)	VDS + LCL161 CI(IC_50_)	DAU + LCL161 CI(IC_50_)	DOX + LCL161 CI(IC_50_)	IDA + LCL161 CI(IC_50_)	ETO + LCL161 CI(IC_50_)	SN-38 + LCL161 CI(IC_50_)	TOPO + LCL161 CI(IC_50_)
**Kelly**	0.53	0.45	0.62	0.96	0.98	1.18	0.88	0.93	1.20
**NB1691luc**	0.53	0.21	0.24	0.65	0.24	0.68	1.11	0.79	1.08
**SH-EP TET21N**	0.46	0.64	0.65	0.72	0.82	1.13	1.10	0.96	0.90
**SK-N-AS**	0.88	0.60	0.46	0.67	0.52	0.62	0.89	0.85	0.41
**SK-N-BE(2)-M17**	0.95	1.11	0.45	0.77	0.63	0.97	0.98	0.77	1.01
**SK-N-SH**	0.88	0.27	0.39	0.83	0.60	0.99	0.99	0.95	1.02
**HGW-1**	0.64	0.50	0.56	1.06	0.66	1.27	0.66	1.12	0.66
**HGW-3**	0.32	0.28	0.26	0.77	0.73	1.05	0.38	0.99	1.14

IC_50_ values for dose-effect curves were determined (**A**). Reduction of IC_50_ by LCL161 is illustrated by red shading (= <10%, 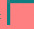 = 10-29%, 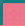 = 30-49%, 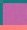 = 50-69%, 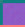 = ≥70%).
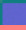

IC_50_ were taken as a basis for the calculation of combination indices (CI) using the Chou-Talay method (**B**).

Range of CI values and correlating intensity of synergism is illustrated by red shading (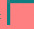 = nearly additive (CI >0.9-1.1) or antagonistic (CI >1.1), = 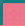 slight synergism (CI = 0.9-0.86), = 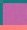 moderate synergism (CI = 0.85-0.71), = 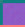 synergism (CI = 0.7-0.31), = strong 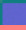 synergism (CI ≤0.3)).

VBL, vinblastine; VCR, vincristine; VDS, vindesine; DAU, daunorubicin; DOX, doxorubicin; IDA, idarubicin; ETO, etoposide; TOPO, topotecan.

### Effects of combination of LCL161 with vinca alkaloids on cellular proliferation

**Figure 2:**
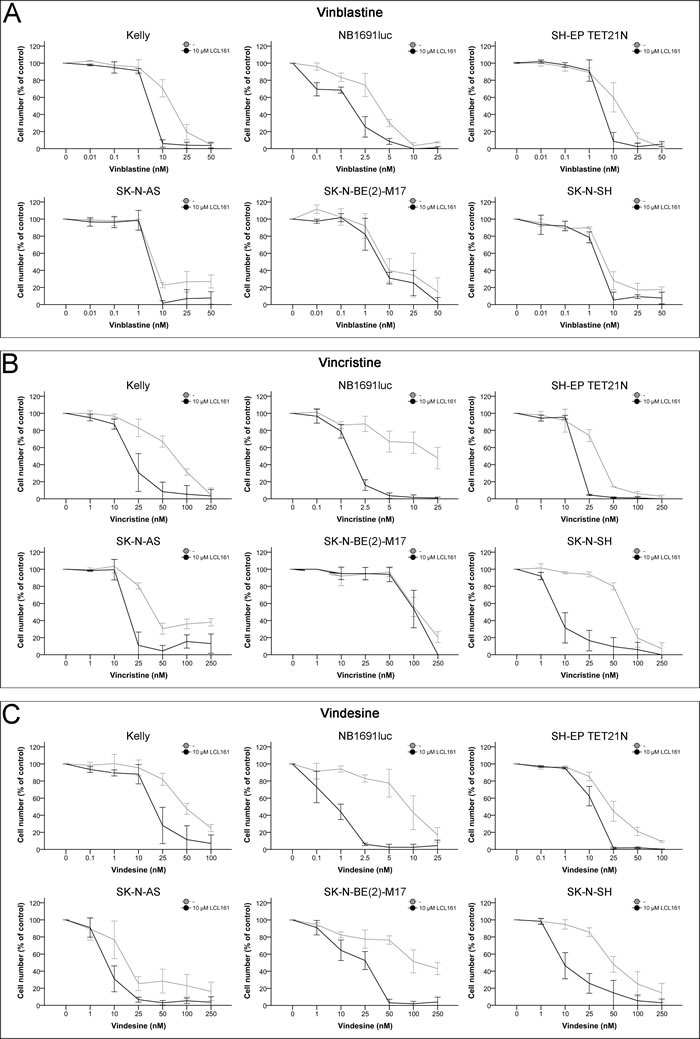
Inhibition of cell proliferation in neuroblastoma cell lines by vinca alkaloids and their combination with LCL161 Cells were treated with the indicated concentrations of vinblastine **A.**, vincristine **B.**, vindesine **C.** or LCL161 **A.**-**C.** and proliferation was determined after 48 h. Proliferation of untreated cells was defined as 100%. Values represent the mean ± SD of three independent experiments.

Treatment of neuroblastoma cell lines with vinca alkaloids (vinblastine, vincristine and vindesine) showed a concentration-dependent inhibition of proliferation (Figure 2 and Table 1A). Vinblastine was the most potent vinca drug with IC_50_ of 3.7-16.4 nM followed by vindesine (IC_50_ 9.1-121 nM) and vincristine (IC_50_ 24.5-117 nM). Combined treatment of LCL161 with each of the vinca alkaloids enhanced the antiproliferative potential of the drugs in a synergistic manner in all cell lines except SK-N-BE(2)-M17 (Figure 2 and Tables 1A+1B). In this cell line only vindesine and LCL161 interacted synergistically.

### Antiproliferative effects of LCL161 co-treatment with anthracyclines

**Figure 3:**
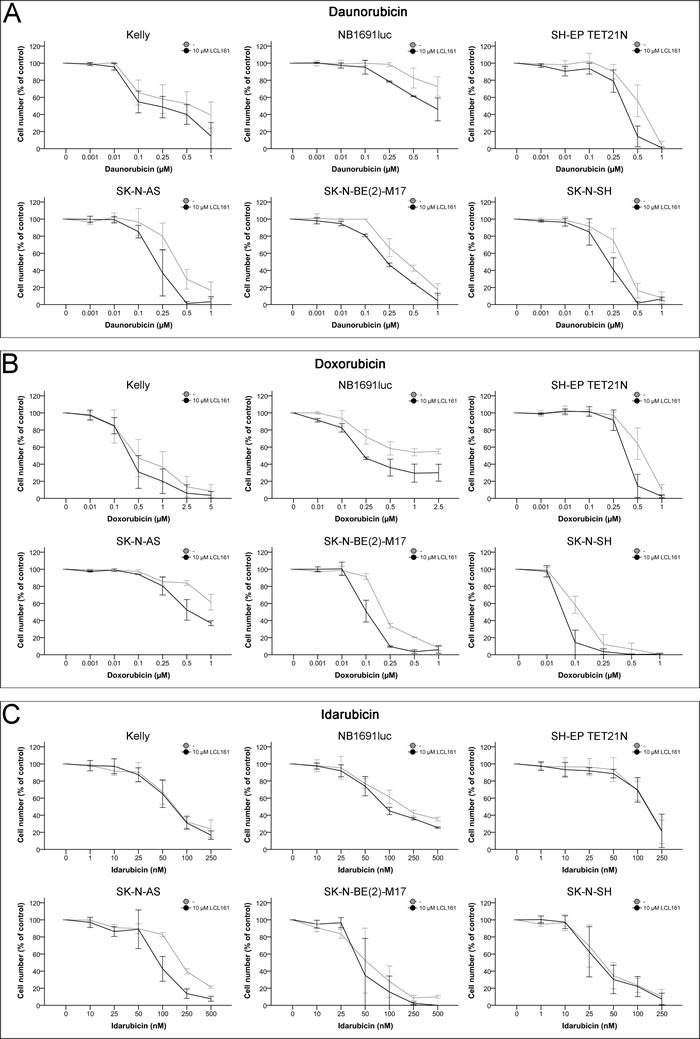
Inhibition of cell proliferation in neuroblastoma cell lines by anthracyclines and their combination with LCL161 Cells were treated with the indicated concentrations of daunorubicin **A.**, doxorubicin **B.**, idarubicin **C.** or LCL161 **A.**-**C.** and proliferation was determined after 48 h. Proliferation of untreated cells was defined as 100%. Values represent the mean ± SD of three independent experiments.

Inhibition of cellular proliferation with anthracyclines disclosed a relative insensibility in comparison to treatment with vinca alkaloids (Figure 3 and Table 1A). Idarubicin induced the strongest decrease of cellular growth with IC_50_ of 42-223 nM. The IC_50_ of the other two anthracyclines were 0.35-1.58 μM for daunorubicin and 0.12-2.58 μM for doxorubicin. Addition of LCL161 enhanced the negative effect on proliferation of daunorubicin and doxorubicin in all cell lines, except Kelly, synergistically (Figures 3A+3B and Tables 1A+1B). Co-treatment of LCL161 with idarubicin showed a synergism only in the cell lines NB1691luc and SK-N-AS (Figure 3C and Tables 1A+1B).

### LCL161 and topoisomerase inhibitor-induced inhibition of proliferation

**Figure 4:**
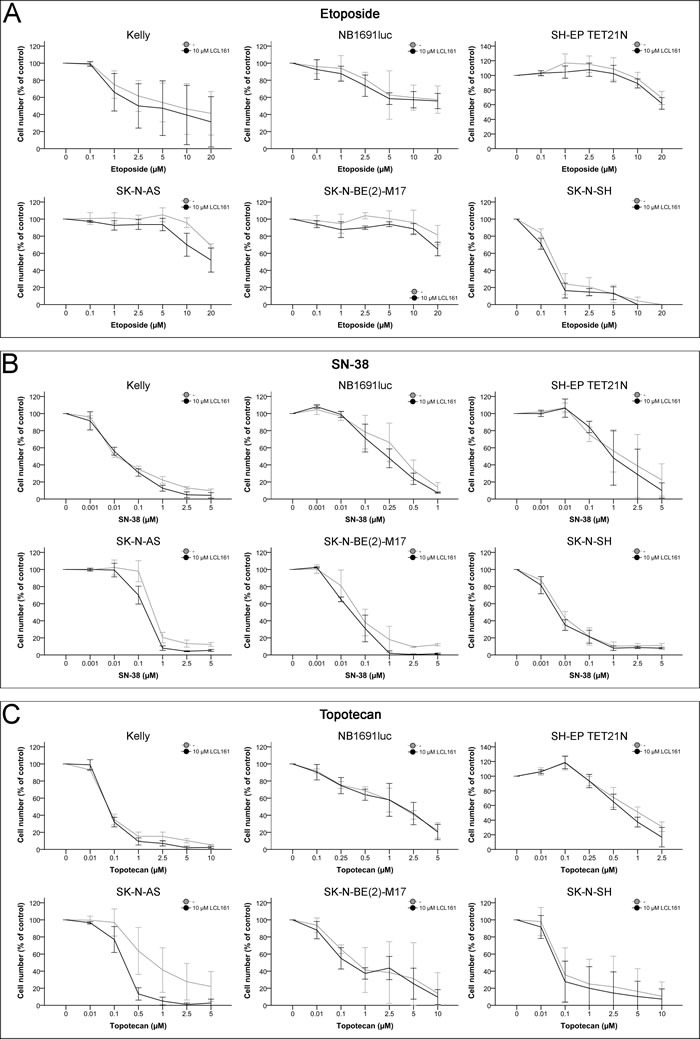
Inhibition of cell proliferation in neuroblastoma cell lines by topoisomerase inhibitors and their combination with LCL161 Cells were treated with the indicated concentrations of etoposide **A.**, SN-38 **B.**, topotecan **C.** or LCL161 **A.**-**C.** and proliferation was determined after 48 h. Proliferation of untreated cells was defined as 100%. Values represent the mean ± SD of three independent experiments.

Five of the six tested cell lines with exception of SK-N-SH (IC_50_ 0.54 μM) were unresponsive against etoposide with IC_50_ of 4.6-29.1 μM (Figure 4A and Table 1A).

In contrast proliferation of neuroblastoma cell lines treated with topoisomerase inhibitors SN-38 (IC_50_ 0.013-1.56 μM) and topotecan (IC_50_ 0.08-1.17 μM) was inhibited to a similar degree (Figures 4B+4C and Table 1A).

Application of LCL161 had marginal effects on the antiproliferative impact of topoisomerase inhibitors. Only in the cell line SK-N-AS addition of LCL161 resulted in a synergistic topotecan-induced inhibition of proliferation (Figure 4C and Tables 1A+1B). In all other cell lines LCL161 was not able to substantially increase the inhibition of proliferation of either etoposide, SN-38 or topotecan leading to additive/antagonistic effects or only slight to moderate synergism (Figure 4 and Tables 1A+1B).

### Antiproliferative effect of LCL161 and chemotherapy in *de novo* NB cell lines

In order to validate that the preceding observations were not restricted to long-term cultivated cell lines proliferation assays were expanded to low passage *de novo* neuroblastoma cell lines HGW-1 and HGW-3. Both were highly sensitive for chemotherapy in general and combination with LCL161 induced effects comparable to that of the established neuroblastoma cell lines (Figures S1 and S2 and Tables 1A+1B). Again, the inhibition of proliferation induced by all vinca alkaloids could be increased by LCL161 leading to (strong) synergism (Figure S2A and Tables 1A+1B). Combinations of LCL161 with anthracyclines or topoisomerase inhibitors elicited a synergism only for single drugs or cells similar to the results obtained before (Figures S2B+S2C and Tables 1A+1B).

### LCL161-mediated sensitization for vinca alkaloid-induced apoptosis

**Figure 5:**
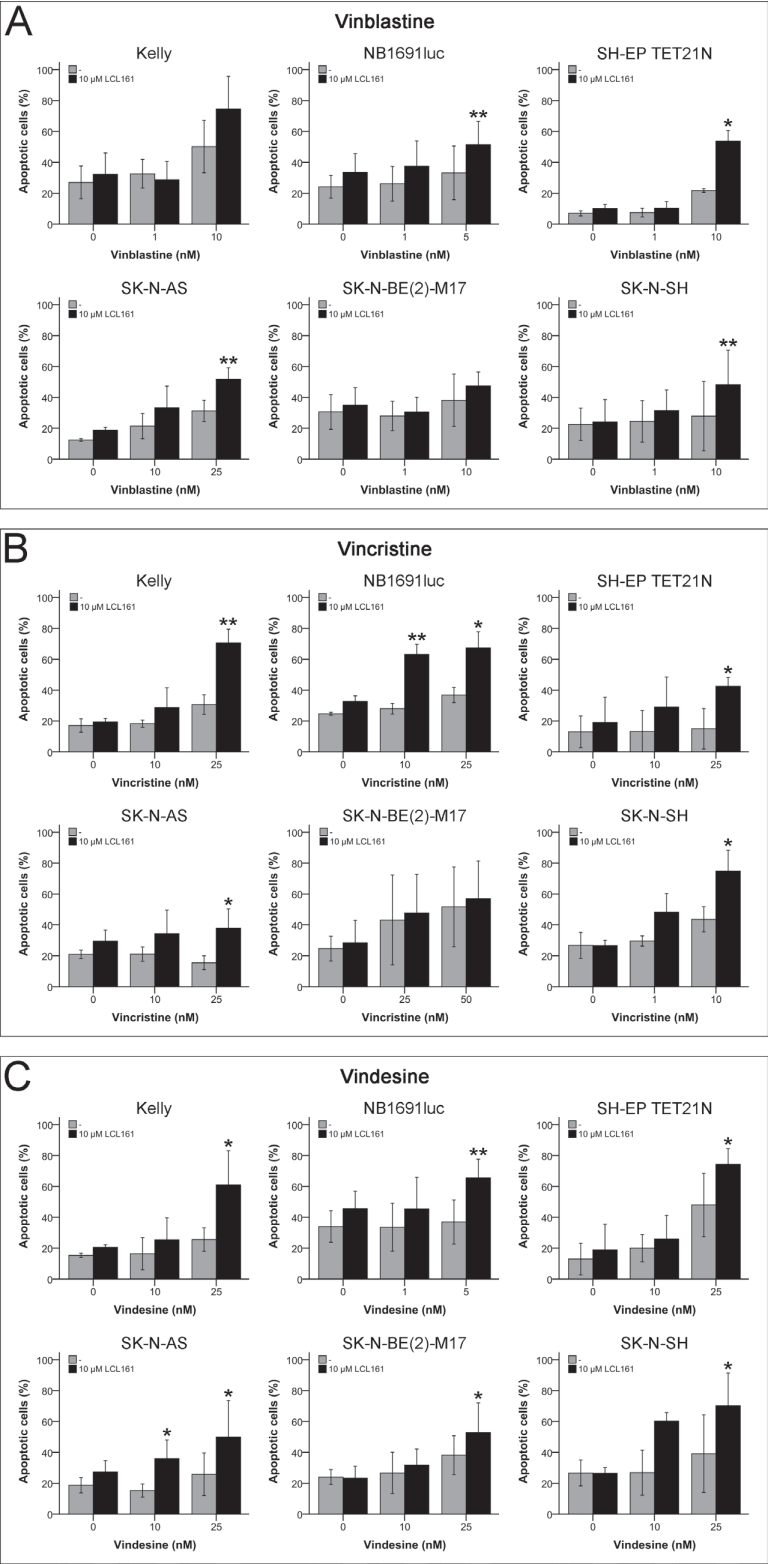
Induction of apoptosis in neuroblastoma cell lines by vinca alkaloids and their combination with LCL161 Cells were treated with the indicated concentrations of LCL161, vinblastine **A.**, vincristine **B.** or vindesine **C.** and the proportion of apoptotic cells was determined by flow cytometry (Annexin V and PI staining) after 48 h. Values represent the mean ± SD of three independent experiments, *; *p* ≤ 0.05, **; *p* ≤ 0.01.

With the intention to shed light on the sensitization for vinca alkaloid-induced inhibition of proliferation by LCL161, cells were analyzed for apoptosis induction using flow cytometry. According to the proliferation experiments combined treatment of LCL161 with vinca alkaloids resulted in a considerable increase of apoptosis in all cell lines except SK-N-BE(2)-M17 (Figures 5A-5C). In four respectively five of the six cell lines a significant sensitization for vinblastine- and vincristine-induced apoptosis by LCL161 was observed (Figures 5A+5B). In line with the proliferation data all cell lines were susceptible for the combined treatment of vindesine and LCL161 leading to a significant increase of apoptotic cells (Figure 5C).

### Apoptosis induction by anthracyclines and LCL161

**Figure 6:**
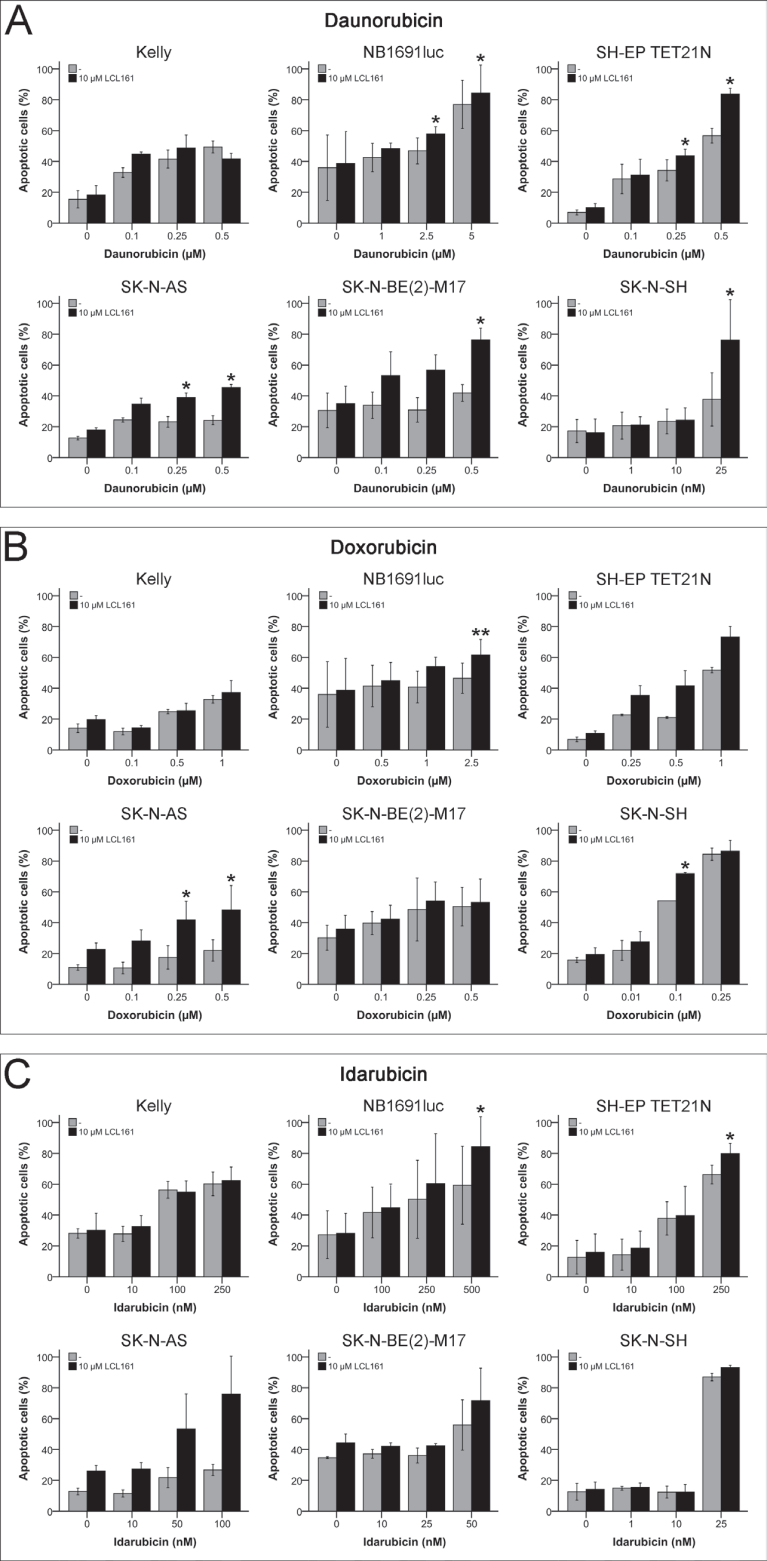
Induction of apoptosis in neuroblastoma cell lines by anthracyclines and their combination with LCL161 Cells were treated with the indicated concentrations of LCL161, daunorubicin **A.**, doxorubicin **B.** or idarubicin **C.** and the proportion of apoptotic cells was determined by flow cytometry (Annexin V and PI staining) after 48 h. Values represent the mean ± SD of three independent experiments, *; *p* ≤ 0.05, **; *p* ≤ 0.01.

Neuroblastoma cell lines were treated with LCL161 in combination with anthracyclines to evaluate the effects on apoptosis induction (Figures 6A-6C). In all cell lines beside Kelly a significant increase in apoptosis was observed by application of LCL161 to daunorubicin correlating with the synergisms observed in the proliferation assays (Figure 6A). A similar pattern was detected for doxorubicin (Figure 6B).

However, unexpectedly no obvious differences in apoptosis were found for doxorubicin and its combination with LCL161 in SK-N-BE(2)-M17 that displayed a synergistic (CI(IC_50_) 0.63) inhibition of proliferation in the combination treatment. Add-on of LCL161 to idarubicin had no significant effect in the cell lines Kelly, SK-N-BE(2)-M17 and SK-N-SH and only a low increase in apoptosis was seen in the cell line SH-EP TET21N (Figure 6C). In SK-N-AS and NB1691luc LCL161 augmented the idarubicin-induced apoptosis thus affirming the synergism detected before (Figure 6C).

**Table 2:** Molecular features of neuroblastoma cell lines and *de novo* cell lines

NB cell line	MYCN	Chromosome 1	ALK	p53
Kelly	>100x amplified[58]	del(1p)	F1174L[48]	wt[59]
NB1691luc	amplified^a^	wt^a^	wt[60]	wt[61]
SH-EP TET21N	wt (conditional MYCN expression)[55, 62]	+1q[62]	F1174L[48]	wt[61]
SK-N-AS	wt[63]	del(1p)[63]	wt[48]	mutated[64]
SK-N-BE(2)-M17	amplified[62]	del(1p), t(1p)[62]	wt[48]	mutated[65, 66]
SK-N-SH	wt[62]	+1q[62]	F1174L[48]	wt[67]
HGW-1	amplified[54]	+1q[54]	unknown	unknown
HGW-3	amplified[54]	del(1p)[54]	unknown	unknown

F1174: The F1174L mutation results in an amino acid substitution at position 1174 in ALK from a phenylalanine (F) to a leucine (L).

a Peter Houghton, personal communication

### Topoisomerase inhibitor-induced apoptosis is only marginally increased by LCL161

**Figure 7:**
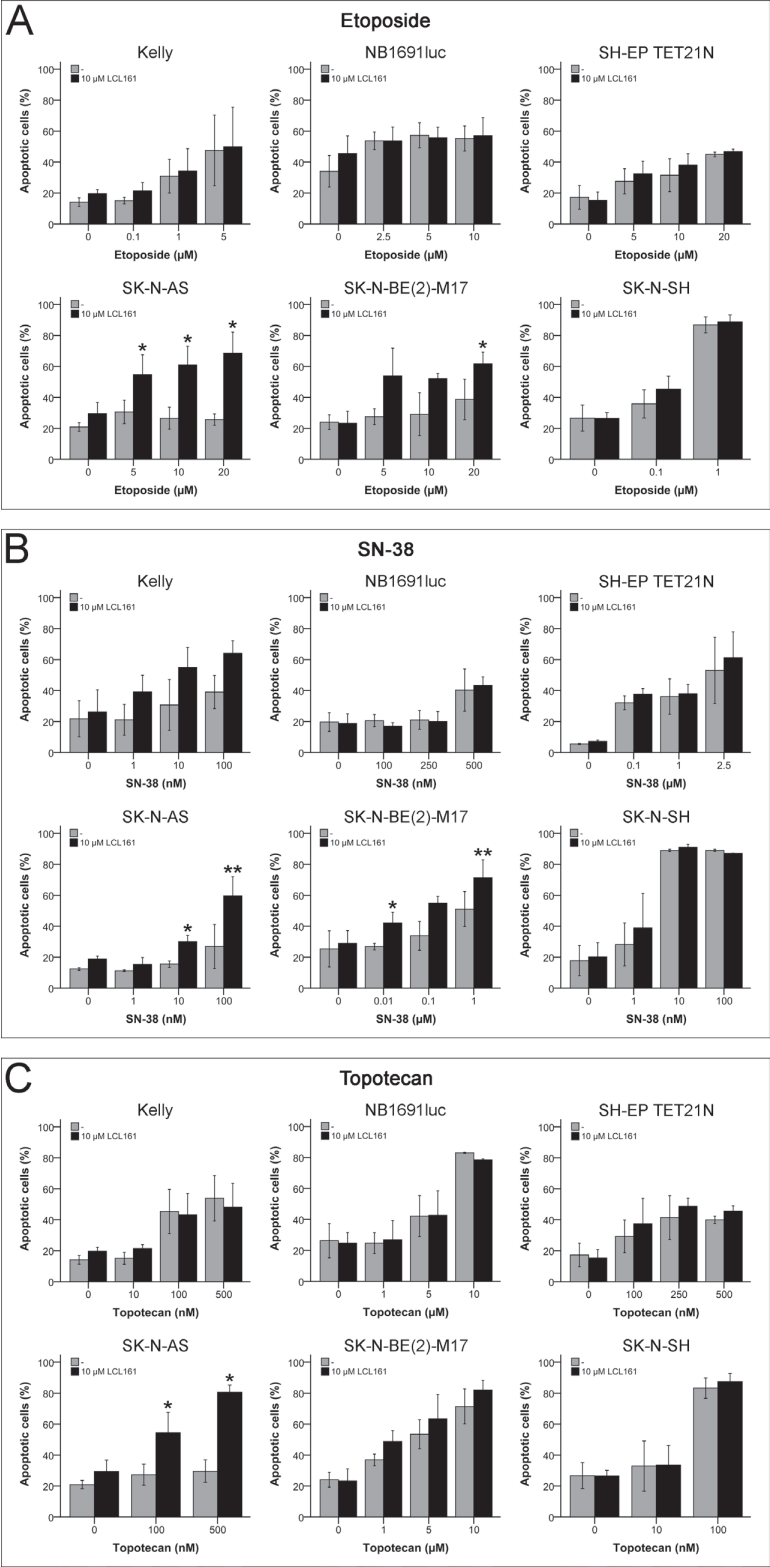
Induction of apoptosis in neuroblastoma cell lines by topoisomerase inhibitors and their combination with LCL161 Cells were treated with the indicated concentrations of LCL161, etoposide **A.**, SN-38 **B.** or topotecan **C.** and the proportion of apoptotic cells was determined by flow cytometry (Annexin V and PI staining) after 48 h. Values represent the mean ± SD of three independent experiments, *; *p* ≤ 0.05, **; *p* ≤ 0.01.

LCL161 had only minor influence on topoisomerase inhibitor-induced inhibition of cellular proliferation of NB cell lines. Therefore, it was of interest if apoptosis induction of this drug class can be increased by combination with SM. Only in the cell line SK-N-AS LCL161 was able to significantly increase the induced apoptosis of all topoisomerase inhibitors in line with the proliferation assays (Figures 7A-7C). In the cell line SK-N-BE(2)-M17 the effects of etoposide and SN-38 were significantly increased by LCL161 for some of the used drug concentrations (Figures 7A+7B).

### ALK activation affects interaction of chemotherapy and LCL161

Molecular aberrations in neuroblastoma including amplification of MYCN, deletions of chromosome arm 1p (del1p) or mutations in p53 or anaplastic lymphoma kinase (ALK) are commonly found in advanced stage or relapsed tumors and are associated with an adverse outcome [21-25]. Therefore, it would be highly relevant to elucidate if and how these alterations (Table 2) could have an influence on sensitization of neuroblastoma cell lines for chemotherapy by LCL161.

For this reason, genetic features of NB cell lines were correlated to combination index (CI) scores reflecting synergistic (CI < 1) or additive/antagonistic (CI ≥ 1) interaction of chemotherapeutic drugs with LCL161 (Tables 1B and 2).

**Figure 8:**
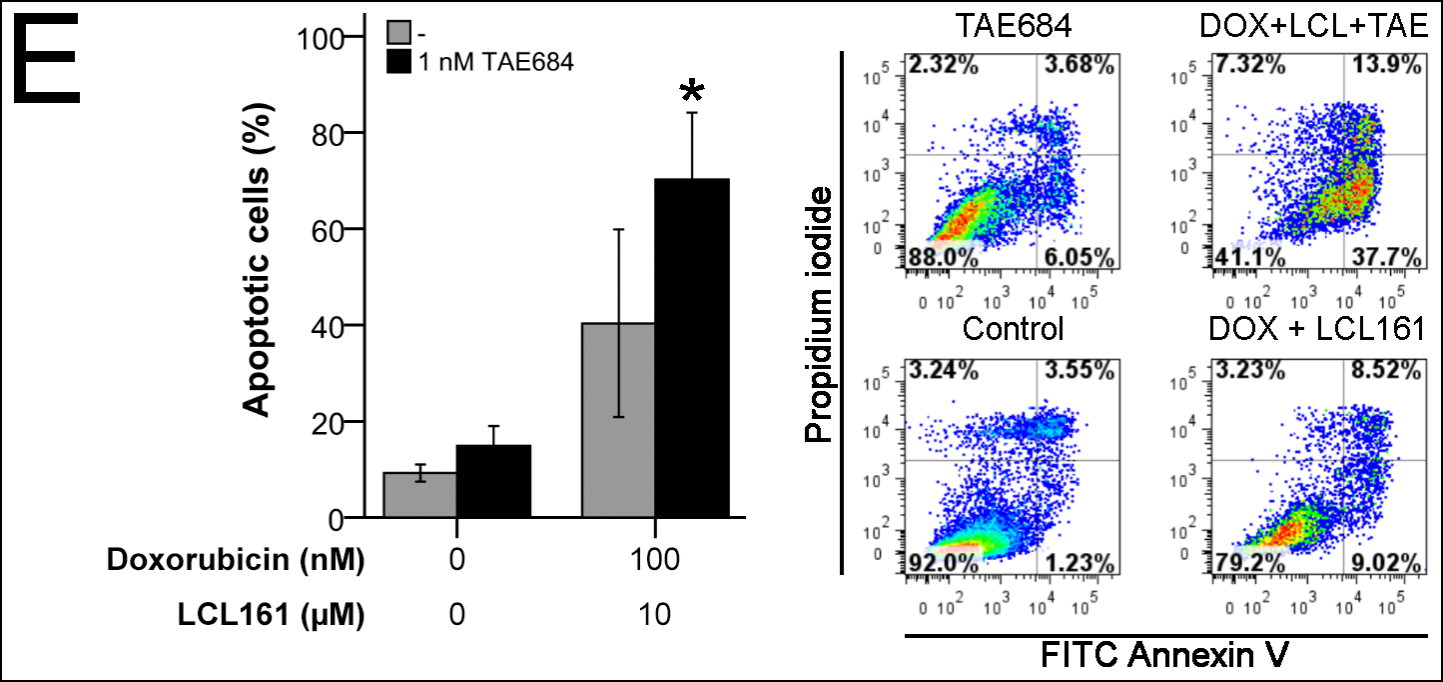
Activating ALK mutation F1174L influences impact of LCL161 and chemotherapy in neuroblastoma ALK status (ALK_wt_ *vs*. ALK_F1174L_) was correlated to combination index (CI) scores reflecting synergistic (CI < 1) or additive/antagonistic (CI ≥ 1) interaction of chemotherapeutic drugs with LCL161 **A.**. Box plots represent the interquartile range (25-75th percentile) and the median value (black line). Whiskers represent the maximum and minimum values, respectively. Proliferation of cells treated with the indicated concentrations of TAE684 was determined after 48h **B.**. Proliferation of untreated cells was defined as 100%. Cells were treated with the indicated concentrations of doxorubicin, LCL161 or TAE684 and inhibition of proliferation was determined after 48h **C.**. Inhibition of proliferation of untreated cells was defined as 0%. IC50 values for dose-effect curves were determined and taken as a basis for the calculation of combination indices (CI) **D.**. SH-EP TET21N cells were treated with the indicated concentrations of doxorubicin, LCL161 or TAE684 and the proportion of apoptotic cells was determined by flow cytometry (Annexin V and PI staining) after 48 h **E.**. Dot plots are representative for the performed analyses. Values represent the mean ± SD of three independent experiments **B.**-**E.,** *; *p* ≤ 0.05.

Using the Mann-Whitney *U* test it became evident that status of MYCN, deletion of chromosome arm 1p or mutated p53 could not account for significant differences between groups (data not shown). In contrast, in cell lines with activating mutation of ALK (ALK_F1174L_) a significantly higher CI than in ALK_wt_ cells was observed for LCL161 and its combination with idarubicin (CI(IC_50_) 1.1 *vs*. 0.76, *p* = 0.05) or SN-38 (CI(IC_50_) 0.94 *vs*. 0.8, *p* = 0.05) (Figure 8A). The largest CI difference (ALK_F1174L_ *vs*. ALK_wt_) was detectable for doxorubicin and LCL161 (CI(IC_50_) 0.8 *vs*. 0.46, *p* = 0.127).

Cells were treated with TEA684 leading to specific inhibition of ALK phosphorylation (Figure S3) and its downstream targets to determine if the aberrant activation of ALK in the NB cell lines Kelly, SH-EP TET21N and SK-N-SH directly influences chemotherapeutic drugs and their interaction with LCL161 [26]. Proliferation of neuroblastoma cell lines (*n* = 6) was efficiently inhibited using a low micromolar range of TAE684 (IC_50_ 0.12-5.37 μM, Figure 8B and Table S1A). No significant differences were observed between ALK_wt_ and ALK_F1174L_ cells. For subsequent experiments TAE684 was used at a concentration of 1 nM, inducing no relevant antiproliferative effects.

Doxorubicin, as one of the standard drugs used in neuroblastoma chemotherapy, was selected for evaluating if ALK inhibition influences the combination of chemotherapy with Smac mimetic LCL161. Cellular proliferation was suppressed by TAE684 in all cell lines leading to enhanced doxorubicin-induced growth inhibition (Figure 8C and Table S1A). Combination of LCL161 with doxorubicin induced a synergistic antiproliferative effect in all cell lines with ALK_wt_. Cell lines harboring mutation ALK_F1174L_ were again less susceptible and showed no synergism (Figures 8C+8D and Tables S1A+S1B).

Simultaneous inhibition of ALK with TAE684 in doxorubicin and LCL161-treated cells had a relevant effect only in ALK_F1174L_ cells (Figure 8C and Table S1A). In all three ALK_F1174L_ cell lines TAE684 caused a strong increase in inhibition of proliferation leading to a significant decrease of the CI(IC_50_) to the synergistic range (Figure 8D and Table S1B). In contrast, TAE684 only marginally influenced the impact of doxorubicin and LCL161 in cells with ALK_wt_ and thus no relevant changes of the CI(IC_50_) were observed (Figures 8C+8D and Tables S1A+S1B).

ALK_F1174L_ SH-EP TET21N cells were most susceptible for TAE684-treatment and were therefore analyzed for apoptosis induction. Treatment with 1 nM TAE684 had only marginal effects on apoptosis. In contrast, the effect of combined doxorubicin and LCL161 was significantly reinforced by TAE684. This was reflected by an increase of early (Annexin V positive) and late apoptotic (Annexin V and propidium iodide positive) cells (Figure 8E).

These findings show that TAE684-induced inhibition of activated ALK (ALK_F1174L_) in neuroblastoma not only is effective to increase the effect of chemo drugs but also is able to synergistically interact with Smac mimetic LCL161. The latter is highly relevant for cells that are resistant for combination of either TAE684 or LCL161 with chemotherapy.

## DISCUSSION

Prognosis for stage 4 high-risk neuroblastoma is still poor albeit significant improvements in therapy were established that supplement high-dose chemotherapy and irradiation with innovative measures as immunotherapy [3]. We have shown that Smac mimetics (SM) are a promising option to overcome chemoresistance [7]. However, general synergism was only achieved with vincristine.

Using a panel of NB cell lines (*n* = 8) and 3 drugs of each class of chemotherapeutics it became apparent that vinca alkaloids generally showed a synergism and significant increase in apoptosis induction with SM LCL161 (Tables 1A+1B and Figures 2, 5 and S2). Vinca alkaloids are antimitotic agents that at high concentrations (e.g. 10-100 nM in HeLa cells) prevent microtubules to polymerize thereby impede the formation of the spindle apparatus necessary for cell division [27].

In contrast, taxanes and other antimitotic agents stabilize microtubules and thus arrest the mitotic process [28].

Further analysis disclosed an activation of the NF-κB pathway by vinca alkaloids and a reduced vinca alkaloid-induced apoptosis if activation of NF-κB pathway was impeded. NF-κB signaling is involved in the regulation of a manifold of cellular processes and has shown to either induce or block apoptosis dependent on the cellular context [29].

Modulation of NF-κB signaling by vinca alkaloids might be one explanation why these class of chemotherapeutics showed such a remarkable potency if combined with Smac mimetics.

Apoptosis induction by SM originally was thought to be solely mediated by direct binding to XIAP and abrogation of its inhibition of caspase activation [10]. Recently the degradation of cIAP-1/−2 and the subsequent activation of NF-κB and induction of TNF-α was proposed as the main molecular effect of SM in tumor cells [18-20]. TNF-α was found to be crucial for synergism of SM with chemotherapy by activating the extrinsic apoptotic pathway [30].

Nevertheless, this model does not seem to be generally valid for neuroblastoma as we could show that neuroblastoma cells were sensitized by SM LBW242 independent of TNF-α [7]. Apoptosis induction in neuroblastoma cell lines using SM BV6 recently has also been demonstrated to be independent of TNF-α signaling [31] Additionally there is evidence that other pathways involving RIP1 could be crucial for SM-mediated sensitization for chemotherapy as well [32]. In neuroblastoma RIP1 has shown to be required for apoptosis induction only for the combination of BV6 and doxorubicin but not for BV6 and vincristine [31]. This was explained by activation of different initial cellular programs by the used drug.

SM LBW242 synergistically increased impact of treatment of neuroblastoma with the anthracycline drug doxorubicin in three of four cell lines [7].

We now showed that the anthracyclines daunorubicin and doxorubicin synergistically interacted with LCL161 in all except one cell line (Tables 1A+1B and Figures 3A+3B). In contrast, with idarubicin only in two cell lines a synergism was detectable (Tables 1A+1B and Figure 3C).

The mechanisms of action that are responsible for cell death induction by anthracyclines are still ambiguous. DNA intercalation leading to inhibition of DNA transcription and replication and more relevant poisoning of topoisomerase II leading to aggregation of damaged DNA, G1/G2 cell cycle arrest and apoptosis were described for anthracyclines to name just a few [33-35].

Cytotoxic eviction of histones and formation of free radicals are other mechanism that obviously are relevant for the anti-tumor effects of anthracyclines [36-38].

The high homology between doxorubicin and daunorubicin, which only differ in a hydroxy group, most likely accounts for the similar effects that were observed in combination with LCL161. Idarubicin is derived from daunorubicin by elimination of a methoxy group resulting in an increased lipophility and better uptake into the cell [39].

SM LBW242-treatment did not significantly increase topoisomerase inhibitor etoposide-induced inhibition of proliferation in neuroblastoma cell lines [7]. Combination of SM LCL161 with etoposide, SN-38 or topotecan now indicated a predominant insensitivity against topoisomerase inhibitors, if compared with clinically reachable concentrations, that can be overcome by LCL161 only in a minority of the cells (Tables 1A+1B and Figures 2, 6 and S2).

Drug resistance during recurrent disease is a major problem in the treatment of cancer [40].

For etoposide it was demonstrated that neuroblastoma cell lines obtained at time of diagnosis were sensitive for etoposide but that resistance obviously was acquired during progressive disease or if disease relapsed [41].

With this in mind it is not surprising that the IC_50_ (4.6-29.1 μM) of almost all (7 of 8) tested NB cell lines were much higher as the achievable plasma steady-state concentration of ~12 μM (7 μg/ml) as only one cell line was established before therapy [42].

A comparable pattern of resistance was seen for SN-38 (IC_50_ 13 nM-1.56 μM *vs*. C­_max_ of 56 nM (22 ng/ml)) [43] and topotecan (IC_50_ 80 nM-1.17 μM *vs*. C­_max_ of 209 nM (88 ng/ml)) [44]. Cross-resistance of SN-38 and topotecan with etoposide is another problem that was observed after intensive chemotherapy and use of topoisomerase inhibitors during recurrent disease was thus not suggested [45].

The use of Smac mimetics in contrast could nevertheless be a promising strategy to overcome topoisomerase inhibitor resistance in some cases. The cell line SK-N-AS disclosed triple resistance against etoposide (IC_50_ 26.7 μM), SN-38 (IC_50_ 0.53 μM) and topotecan (IC_50_ 0.78 μM) but all drugs showed a synergism with LCL161. This synergism lowered the IC_50_ to 20.4 μM for etoposide, to 0.38 μM for SN-38 and to 0.22 μM for topotecan. As these concentrations are only clinically achievable for topotecan, it would however have relevant clinical implications to find molecular markers that are causal for these findings.

Systematical analysis of susceptibility of different drug classes for LCL161 revealed a so far unknown association with mutation of ALK (ALK_F1174L_). Interestingly, other molecular aberrations like MYCN status did not influence impact of LCL161. Point mutations activating the ALK tyrosine kinase domain were found in familial and sporadic neuroblastomas with high frequency [26, 46-48].

In neuroblastoma the most common and aggressive modified ALK variant (ALK_F1174L_) is necessary for cellular proliferation and survival [26]. Furthermore, ALK_F1174L_ targeted expression has been demonstrated to be tumorigenic in mice inducing neuroblastomas that resemble the human disease [49]. Transgenic ALK_F1174L_ mice were dependent on ALK_F1174L_ activity as treatment with ALK inhibitor TAE684 induced a complete tumor regression.

Consequently, we could show that inhibition of ALK using TAE684 induces a significant decrease in cellular proliferation of neuroblastoma cell lines (Figure 8B).

For the first time we demonstrated that SM LCL161 positively interacted with TAE684 (Figures 8C+8D), while exhibiting minor effects only together with doxorubicin in ALK_F1174L_ cells (Figures 3B and 8C+8D).

Combined inhibition of ALK using TAE684 however was able to overcome LCL161-resistance leading to a synergistic inhibition of cell growth (Figures 8C+8D).

In contrast, in ALK_wt_ cells the effects of LCL161 and doxorubicin were not significantly increased by TAE684.

This is in line with other findings that showed a stronger impact of TAE684 on ALK_F1174L_ than ALK_wt_ [26, 50]. Activated ALK controls a manifold of pathways including phosphatidylinositol-3 kinase (PI3K) signaling [51]. Activated PI3K in turn activates Akt that is directly involved in regulation of apoptosis. XIAP phosphorylation by Akt protects XIAP from cisplatin-induced ubiquitination and degradation thus preventing apoptosis induction [52]. In pancreatic cancer cells, which were resistant for SM AZD5582, knockdown of Akt using RNAi sensitized them for AZD5582 [53]. Moreover, extent of AZD5582-induced apoptosis was dependent on the expression levels of p-Akt and p-XIAP.

Taken together a hypothesis evolves that would give a molecular basis for the observed synergism of TAE684 and LCL161 in ALK_F1174L_ neuroblastoma. Elevated XIAP levels and XIAP stabilization by ALK_F1174L_/PI3K/Akt-mediated phosphorylation would potentially confer a strong resistance against apoptotic stimuli. On this background, inhibition of ALK by TAE684 would destabilize XIAP by preventing its phosphorylation by Akt. LCL161-treatment would disturb XIAP interaction and inhibition of caspases. Both TAE684 and LCL161 have the potential to induce apoptosis but are especially effective in supporting other apoptotic stimuli. In combination, their impact on XIAP is further increased leading to a considerable gain of chemotherapy.

In summary, ALK inhibition together with SM LCL161 could be a highly effective option to significantly augment chemotherapy by synergistically suppressing inhibitor of apoptosis proteins.

## MATERIALS AND METHODS

### Cell lines and cell culture

Neuroblastoma cell lines Kelly, NB1691luc, SH-EP TET21N, SK-N-AS, SK-N-BE(2)-M17 and SK-N-SH as well as recently established *de novo* cell lines HGW-1 and HGW-3 (kindly provided by Holger Lode; University Medicine Greifswald) [54] were used. The SH-EP TET21N system is a conditional, tetracycline-regulated MYCN expression system established in the SH-EP neuroblastoma cell line [55]. NB1691luc are luciferase-expressing, zeocin-resistant NB1691 cells [56]. Neuroblastoma cells were maintained in RPMI 1640 or IMDM medium (HGW-1 and HGW-3) supplemented with 10-20% fetal bovine serum (both Life Technologies, Darmstadt, Germany), penicillin/streptomycin (10.000 U/ml / 10.000 g/ml, Biochrom, Berlin, Germany) and zeocin (100 μg/ml; NB1691luc). All cells were cultivated at 37°C, 5% CO_2_-atmosphere and a relative humidity of 95%.

### Chemical compounds, biological reagents and drugs

Novartis Pharma generously provided Smac mimetic LCL161. Cytostatic drugs daunorubicin, doxorubicin, etoposide, idarubicin, topotecan, SN-38 (active metabolite of irinotecan), vinblastine, vincristine and vindesine were obtained from Sigma-Aldrich (Munich, Germany). NVP-TAE684 was purchased from Selleckchem (Munich, Germany). Working solutions were prepared by dilution of drugs with medium or ddH_2_O to designated concentrations.

### Protein extraction and Western blot analysis

Cell lysates were prepared with radioimmunoprecipitation assay (RIPA) buffer (Sigma-Aldrich) supplemented with Complete protease inhibitor cocktail and PhosSTOP - Phosphatase Inhibitor Cocktail (Roche). Standard procedures for Western blotting were followed using the following primary antibodies: cIAP-1 (AF8181; R&D), XIAP (610762; BD Biosciences) and *β*-actin (A1978; Sigma-Aldrich). Specific protein bands were visualized using IRDye 680RD or 800CW secondary antibodies (LI-COR) and LI-COR Odyssey infrared imaging system.

### Total ALK and Phospho-ALK ELISA

Expression of total ALK and phospho-ALK was quantified using PathScan^®^ Total ALK and Phospho-ALK (Tyr1604) Chemiluminescent Sandwich ELISA Kits (7084C and 7020C, Cell Signaling) according to the manufacturer's protocols. Protein concentrations of lysates were determined using Bio-Rad Protein Assay and bovine serum abumin as protein standard.

### Cellular proliferation assays

Proliferation assays were performed with Cell Proliferation Reagent WST-1 (Roche, Grenzach, Germany) according to the manufacturer's protocol. Cells were seeded in culture medium in 96-well plates to adhere overnight. Cytostatic drugs and LCL161 were added to the cells for an incubation period of 48 hours. Following incubation with WST-1 for 2 hours, absorbance was measured with an ELISA reader. IC_50_ values were determined using in-house software (Microsoft Excel).

### Detection of apoptosis by flow cytometry

Cells were seeded in cell culture medium in 24-well plates to adhere overnight. Cells were treated with the indicated reagents for 48 h. Cells were harvested, washed twice with PBS and resuspended in Annexin V binding buffer (10 mM Hepes, 140 mM NaCl, and 0.25 mM CaCl_2_). Apoptosis was detected by Annexin V-FITC (556419; BD Pharmingen, Heidelberg, Germany) and propidium-iodide (PI) (1 mg/ml in ddH_2_O; Invitrogen, Darmstadt, Germany) staining and flow cytometry.

### Statistical analysis

Combination indices (CI) for cellular proliferation assays were calculated using the Chou-Talay method to determine synergism (CI ≤ 0.9), additivity (CI > 0.9-1.1), or antagonism (CI > 1.1), if combining LCL161 with cytostatic drugs [57]. The CI was calculated according to the classic isobologram equation: CI = (*d1/D1*) + (*d2/D2*), where D1 and D2 represent the required doses of drug 1 and 2 to produce x% effect and d1 and d2 the required doses of drug 1 and 2 to produce the same effect if used in combination. Statistical significance of differences between experimental groups was determined using the Student *t*-test or the Mann-Whitney *U* test. A two tailed *p*-value ≤ 0.05 was regarded as significant.

## SUPPLEMENTARY MATERIALS TABLES FIGURES


