# Supplementary material for: Smac mimetic LCL161 supports neuroblastoma chemotherapy in a drug class-dependent manner and synergistically interacts with ALK inhibitor TAE684 in cells with ALK mutation F1174L
Source: Oncotarget. 2016 Sep 15;7(45):72634–53. doi: 10.18632/oncotarget.12055 (PMC5341933; doi:10.18632/oncotarget.12055)
Supplement: Supplementary file 1 [file oncotarget-07-72634-s001.pdf]

Smac mimetic LCL161 supports neuroblastoma chemotherapy in a drug class-dependent manner and synergistically interacts with ALK inhibitor TAE684 in cells with ALK mutation F1174L

Supplementary Material

Table S1. ALK inhibition with TAE684 significantly increases inhibition of cell proliferation induced by combination of doxorubicin with LCL161 in neuroblastoma cell lines with ALK mutation F1174L.

Table S1A

	DOX IC_{50} (μM)					
**LCL161** (10 μM)	-	+	-	+		
**TAE684** (1 nM)	-	-	+	+	**LCL161** IC_{50} (μM)	**TAE684** IC_{50} (μM)
**Kelly**	.23	.19	.24	.13	49	5.37
**SH-EP TET21N**	.22	.19	.19	.07	73	0.12
**SK-N-SH**	.38	.28	.38	.16	56	1.12
**NB1691luc**	1.09	.6	.67	.29	69	0.74
**SK-N-AS**	.97	.23	.79	.15	78	0.83
**SK-N-BE(2)-M17**	.3	.18	.23	.16	59	3.73

Table S1B

	**DOX + LCL161** $CI(IC_{50})$	**DOX + LCL161 + TAE684** $CI(IC_{50})$
**Kelly**	1.05	0.75
**SH-EP TET21N**	0.98	0.50
**SK-N-SH**	0.92	0.61
**NB1691luc**	0.70	0.57
**SK-N-AS**	0.36	0.32
**SK-N-BE(2)-M17**	0.78	0.84

Table S1. **ALK inhibition with TAE684 significantly increases inhibition of cell proliferation induced by combination of doxorubicin with LCL161 in neuroblastoma cell lines with ALK mutation F1174L.** IC_{50} values for dose-effect curves were determined (**A**). IC_{50} were taken as a basis for the calculation of combination indices (CI) using the Chou-Talay method (**B**). Range of CI values and correlating intensity of synergism is illustrated by grey shading (□ = nearly additive (CI >0.9-1.1) or antagonistic (CI >1.1), ■ = slight synergism (CI = 0.9-0.86), ■ = moderate synergism (CI = 0.85-0.71), ■ = synergism (CI = 0.7-0.31), ■ = strong synergism (CI ≤0.3)). DOX, doxorubicin.

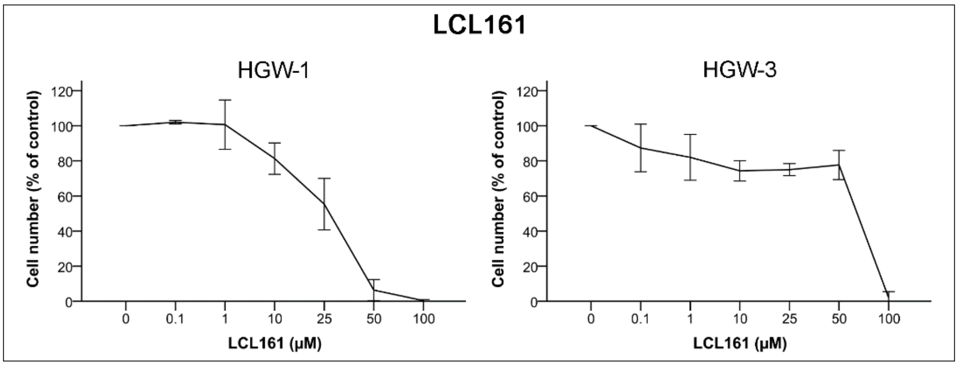

Figure S1. Inhibition of cell proliferation in *de novo* neuroblastoma cell lines by LCL161. Cells were treated with the indicated concentrations of LCL161 and proliferation was determined after 48h. Proliferation of untreated cells was defined as 100 %. Values represent the mean ± SD of three independent experiments.

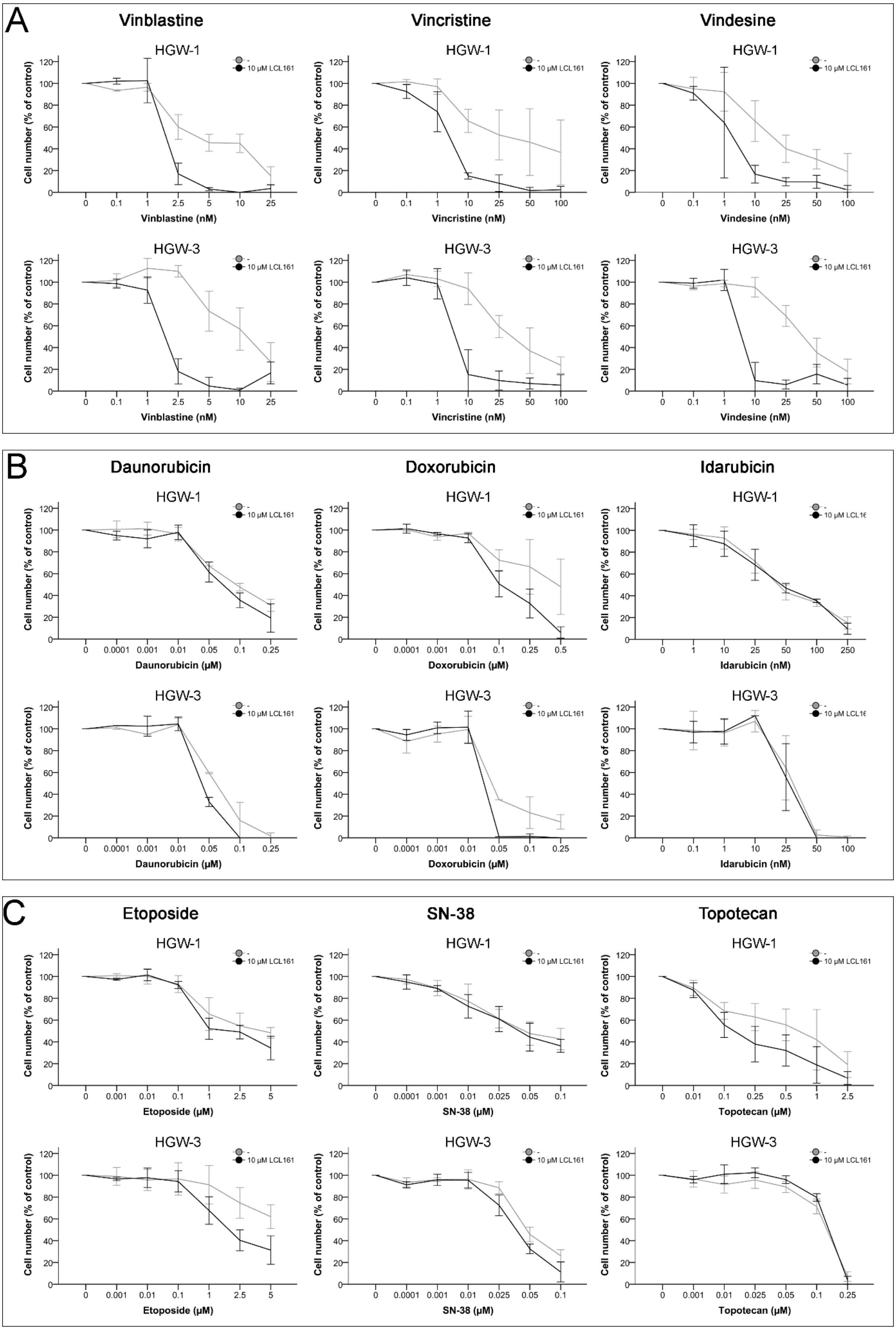
A
Vinblastine
Vincristine
Vindesine
HGW-1
HGW-3
10 µM LCL161
Cell number (% of control)
Vinblastine (nM)
Vincristine (nM)
Vindesine (nM)
B
Daunorubicin
Doxorubicin
Idarubicin
Daunorubicin (µM)
Doxorubicin (µM)
Idarubicin (nM)
C
Etoposide
SN-38
Topotecan
Etoposide (µM)
SN-38 (µM)
Topotecan (µM)

Figure S2. Inhibition of cell proliferation in *de novo* neuroblastoma cell lines by vinca alkaloids, anthracyclines or topoisomerase inhibitors and their combination with LCL161. Cells were treated with the indicated concentrations of vinblastine/vincristine/vindesine **A.**, daunorubicin/doxorubicin/idarubicin **B.**, etoposide/SN-38/topotecan **C.** or LCL161 (**A.-C.**) and proliferation was determined after 48 h. Proliferation of untreated cells was defined as 100 %. Values represent the mean ± SD of three independent experiments.

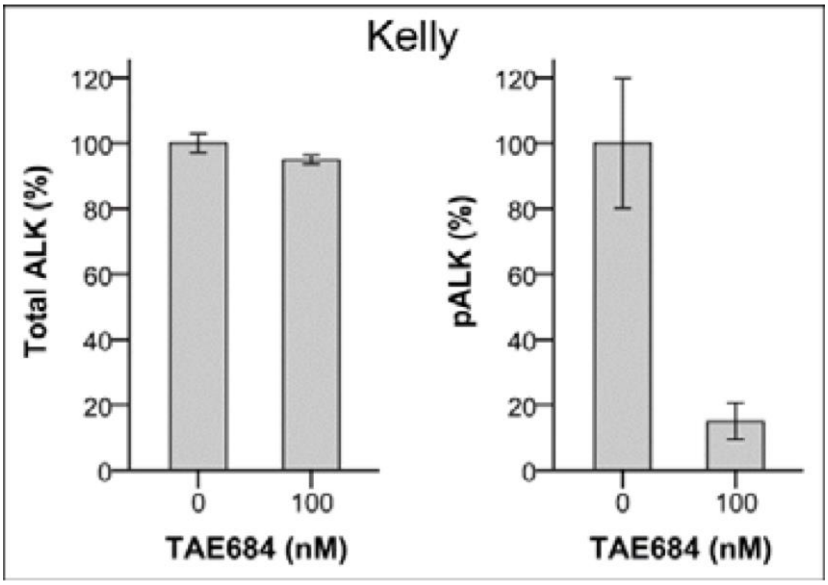

Figure S3. Effect of ALK inhibitor TAE684 on protein levels of ALK and pALK in Kelly neuroblastoma cell line. Expression of total ALK and pALK was detected by ELISA in Kelly cells treated for 6 h with 100 nM TAE684. Total protein content of lysates was used to normalize determined expression values. Values represent the mean ± SD of duplicates.
